# Machine learning based detection of covert communications under jamming interference

**DOI:** 10.1038/s41598-026-53830-8

**Published:** 2026-05-20

**Authors:** E. Esmaili, R. Hajizadeh, M. Forouzesh

**Affiliations:** 1 Amol University of Special Modern Technologies, Amol, Iran; 2 Machine Learning and Deep Learning Laboratory, Faculty of Engineering Modern Technologies, Amol University of Special Modern Technologies, Amol, Iran; 3 Faculty of Engineering Modern Technologies, Amol University of Special Modern Technologies, Amol, Iran

**Keywords:** Covert communication, Feature extraction, Pattern recognition, Physical-layer security, Signal classification, Engineering, Mathematics and computing

## Abstract

In large-scale Internet of Things (IoT) networks, detecting covert communications—hidden transmissions that evade monitoring—is essential to prevent misuse of wireless infrastructure. However, challenges such as fading, noise, jamming interference, and unpredictable traffic complicate reliable detection by a monitoring node (Willie). This paper introduces a hybrid analytical-machine learning (ML) framework for robust detection of covert signals under jamming and Rayleigh fading conditions. An analytical energy detection model is first derived to compute false alarm and missed detection probabilities, establishing a baseline and informing Monte Carlo simulations for dataset generation. The real and imaginary components of simulated complex baseband signals are extracted as features, enabling supervised training of Decision Tree (DT) and Random Forest (RF) classifiers without prior knowledge of transmit powers. Evaluations demonstrate that both ML models outperform the analytical benchmark, with RF achieving a 26.8% reduction in total detection error at a distance of d = 1 km. Further assessments across varying transmitter and jammer power levels confirm the framework’s robustness in interference-limited environments. By integrating theoretical modeling for data credibility with data-driven ML for adaptive classification, this approach provides a scalable, power-agnostic solution for securing real-world wireless networks against covert threats.

## Introduction

Covert communication, also referred to as low probability of detection (LPD), has emerged as a crucial area of research in the context of modern communication systems, particularly in environments where security and secrecy are paramount^1,2^. The concept behind covert communication is to transmit information in such a way that it remains undetected by unintended receivers, often referred to as adversaries or eavesdroppers^3^. Unlike traditional secure communication methods that focus on ensuring confidentiality by encrypting the transmitted data, covert communication focuses on concealing the existence of the transmission itself^4^.

This field commonly employs a canonical terminology to describe the involved parties: a transmitter (Alice) who aims to send a covert message to an intended receiver (Bob), while a passive warden or adversary (Willie) attempts to detect the existence of this transmission. Furthermore, a friendly jammer is often introduced as a cooperative entity to generate artificial noise, specifically designed to confuse Willie without significantly disrupting Bob’s reception.

The covert communication paradigm has gained substantial interest in recent years due to increasing concerns over physical-layer security in wireless networks^5–7^. Use cases such as tactical operations, emergency response infrastructures, and the rapidly expanding Internet of Things (IoT) ecosystem have further intensified the demand for stealthy transmission techniques^8^. These techniques enable sensitive information to be conveyed without revealing the very existence of communication, thereby preserving operational confidentiality even in the presence of powerful adversaries^9^. However, in large-scale IoT deployments, this same capability can also be misused by malicious or compromised devices to perform unauthorized or covert activities. This risk underscores the importance of effective monitoring mechanisms capable of reliably detecting hidden transmissions, ensuring that IoT networks remain secure, trustworthy, and protected against unintended or harmful behavior^10^.

Covert communication presents a fundamental trade-off between reliability and covertness. Increasing transmission power can improve reliability at the Bob but simultaneously increases detectability by the Willie^11^. As the power increases, so does the likelihood of detection, which ultimately compromises the very objective of covert communication^12^. This inherent trade off means that covert communication systems must operate under stringent power constraints, where the total detection error probability or other divergence based secrecy metrics often define the system’s performance limits^13^. This power-constrained optimization challenge is also central to the design of covert systems in advanced network architectures, such as UAV-assisted terahertz networks^14^. Hence, maintaining a balance between ensuring reliable communication while minimizing detection risk is a key challenge in the design of these systems^15^.

In practical covert communication environments, the presence of jamming and interference plays a crucial role in determining both the detectability and reliability of the transmission^16^. A jammer can either serve as a disruptive source that increases detection uncertainty at the Willie or as an intentional cover signal used to mask Alice’s transmission activity. Several studies have investigated the dual effect of jammers, showing that while excessive jamming may degrade Bob’s communication reliability, a carefully controlled jammer can effectively enhance covertness by increasing the Willie’s false alarm probability (${P}_{FA}$)^17–21^.

In practical wireless environments, signal propagation is affected by multiple impairments, including fading, noise, and external jamming. Fading arises from multipath reflections and time varying channel conditions, causing random fluctuations in the received signal’s amplitude and phase and thereby introducing additional uncertainty in the detection process. When combined with additive noise and intentional jamming, these effects lead to significant variability in the received signal statistics, making it increasingly difficult for the Willie to distinguish between Alice’s active and silent states. Such complex and dynamic channel conditions highlight the necessity of adopting more adaptive and data driven detection mechanisms that can maintain reliable performance under non-ideal environments^22^.

Traditional detection methods in covert communication systems primarily rely on energy detection, where a threshold is established for detecting the presence or absence of Alice’s transmission. These methods assume idealized conditions such as stable channel properties and negligible noise or interference^23^. However, real world wireless environments are characterized by non-ideal factors, including multipath fading, time varying noise, and co channel interference, all of which significantly degrade the performance of detection schemes^24^. These conditions result in the failure of traditional detection approaches to capture the complex and dynamic variations in the received signals, which limits their applicability in realistic covert communication scenarios.

Given these limitations, the need for data driven detection methods has become more pronounced. In recent years, machine learning (ML) has increasingly influenced many domains by improving decision making and system efficiency through data driven insights^25^. Machine learning has emerged as a promising alternative, providing a powerful framework for addressing the nonlinearities and dynamic nature of wireless channels. Unlike traditional methods that rely on predefined models and assumptions, machine learning algorithms are capable of learning complex patterns from empirical data, which makes them particularly suited for detecting covert transmissions under uncertain and time varying conditions^26^. ML techniques can capture intricate relationships between the signal features and the transmission state, enabling detectors to operate effectively even when traditional fixed threshold methods fail.

### Related work and state-of-the-art approaches

The detection of covert communication has been investigated from multiple perspectives in the literature, with existing approaches generally classified into analytical, statistical, and data-driven methods.

A significant portion of prior work is based on analytical detection frameworks, where the warden performs hypothesis testing using energy-based metrics. In these approaches, the received signal energy is compared against a predefined threshold to distinguish between transmission hypotheses. These methods are well established in the literature and provide tractable mathematical formulations^4^. However, they rely on strong assumptions, including perfect knowledge of system parameters such as transmit power, jammer power, and noise variance, which limits their effectiveness in realistic environments.

To address this limitation, several studies have proposed theoretical optimization techniques, where the detection threshold is optimized to minimize performance metrics such as the total detection error probability^27^. While these approaches improve analytical performance, they still depend on accurate statistical modeling and prior knowledge, making them sensitive to channel uncertainty and dynamic interference.

In addition, sequential detection methods, such as change-point detection and CUSUM-based approaches, have been explored to incorporate temporal dynamics into the detection process^28^. These techniques enhance sensitivity to signal variations over time but introduce additional complexity and remain fundamentally model-based, limiting their robustness under highly uncertain channel conditions.

More recently, machine learning techniques at the network level have been applied to detect covert behavior using traffic-based features such as packet timing, size, and protocol patterns^29^. While these methods offer high adaptability and reduced dependence on prior knowledge, they are not directly applicable to physical-layer covert communication, as they do not utilize raw signal representations.

Some studies have also explored machine learning-based detection frameworks using engineered features, where statistical descriptors of signals are used for classification tasks^25^. These approaches improve adaptability compared to analytical methods; however, their performance is constrained by feature design and may not fully capture the complex structure of baseband signals under fading and jamming.

A structured comparison of these approaches is provided in Table 1, highlighting their differences in terms of knowledge requirements, feature representation, robustness, and adaptability.

**Table 1:** Comparison of detection paradigms for covert communication under uncertainty.

Detection paradigm	Representative approach	Knowledge requirement	Feature type	Robustness to fading & jamming	Adaptability	Limitation
Analytical detection	Energy Detection (Threshold-based)^4^	High	Scalar energy	Low	Low	Fails under uncertainty and dynamic channels
Theoretical optimization	Optimal Threshold / DEP minimization^27^	High	Statistical metrics	Low–Moderate	Low	Assumes perfect channel knowledge
Sequential detection	CUSUM / Change-point methods^28^	Moderate–High	Time-series statistics	Moderate	Moderate	Complex, still model-based
ML-based (Network-level)	Random Forest (RF) / XGBoost on traffic data^29^	Low	Traffic features	Moderate	High	Not applicable to physical-layer signals
ML-based (general covert detection)	Classification-based ML approaches^25^	Low	Engineered features	Moderate–High	High	Limited signal-level modeling
Proposed work	Random Forest / Decision Tree (DT) Raw real (in-phase) and imaginary (quadrature) samples (complex baseband)	Low (Power-agnostic)	Raw complex-baseband samples (real and imaginary parts)	High	High	Requires training data

### Motivation and innovation from the Willie’s perspective

While the majority of the literature on covert communication primarily focuses on designing transmission schemes for legitimate users (Alice and Bob) to maximize covertness, the present work adopts a fundamentally different standpoint by analyzing the problem from the vantage point of the Willie. This shift in perspective is critical, as it addresses the detection capabilities of an adversary in realistic and complex environments a facet that has received comparatively less attention in prior studies. Although previous research has extensively investigated the impact of channel impairments such as multipath fading, friendly jamming, and noise through theoretical modeling, these approaches often rely on simplified assumptions and closed-form asymptotic expressions that assume perfect knowledge of system parameters (e.g., transmit powers, noise variance). In practice, however, wireless environments are highly dynamic, and acquiring such precise information at the warden’s receiver is challenging, limiting the applicability of purely theoretical models.

The primary innovation of this study lies in bridging the gap between abstract theoretical models and practical signal processing. Unlike conventional studies that limit their analysis to deriving probability-of-error formulas based on channel statistics, we approach the problem from a signal-centric viewpoint. Specifically, we directly process the complex baseband signal components—extracting the in-phase and quadrature data—to construct an ML-based detection framework that mirrors the theoretical model but operates on actual signal observations. By leveraging supervised learning algorithms, specifically Decision Tree and Random Forest, we enable the detection system to learn intricate patterns embedded in the signal structure that correspond to the underlying theoretical derivations. This data-driven methodology represents a significant methodological shift, as it translates the covert communication detection problem from a purely theoretical hypothesis test into a robust, signal-based classification task suitable for real-world implementation.

Moreover, unlike traditional energy detection that rely on fixed thresholds and precise knowledge of transmit powers or noise statistics, the proposed framework operates in a power-agnostic manner by learning directly from the Raw real and imaginary samples. This property is particularly valuable in large-scale IoT environments, where devices operate with heterogeneous power levels, sporadic transmission patterns, and unpredictable interference sources. In such networks, maintaining accurate parameter estimates for analytical detectors is often impractical. By contrast, the proposed ML-based detector learns adaptive, nonlinear decision boundaries directly from observed data, making it well suited for real-world monitoring scenarios where Willie observes a mixture of legitimate IoT traffic and potentially covert transmissions.

In summary, the main contributions of this work are as follows:

- A machine learning–based warden framework that dramatically improves covert communication detection performance compared to conventional energy detection.
- A power-agnostic detection approach using raw samples consisting of real and imaginary components, eliminating reliance on precise knowledge of transmit powers and noise statistics.
- Reformulation of covert detection as a supervised classification task, enabling adaptive, nonlinear decision boundaries that capture complex signal structures.
- A method for extracting and preparing feature vectors for each time slot by sampling the real and imaginary components of the received baseband signal.
- Comprehensive robustness evaluation across both distance (path loss) and power (transmitter/jammer) dimensions, confirming performance under diverse channel conditions.

Overall, the presented framework provides an effective bridge between theoretical modeling and data-driven learning, offering a scalable, adaptive, and power-agnostic solution for covert communication detection in realistic wireless environments.

The remainder of this paper is organized as follows. "System and channel model" section introduces the system and channel models, describing the wireless environment and the interaction among Alice, Bob, Willie, and the friendly jammer under fading and noise conditions. "Detection errors and optimal threshold" section presents the analytical formulation of the energy detection process and derives the theoretical expressions for false alarm and missed detection probabilities (${P}_{MD}$). "The proposed machine learning based covert communication detection" section describes the proposed machine learning based detection framework, including signal generation, dataset preparation, and model training using Decision Tree and Random Forest classifiers. "Experiments and results" section reports the experimental setup and performance results obtained from both analytical simulation comparisons and ML based evaluations. Finally, "Conclusion" section concludes the paper and summarizes the key findings.

## System and channel model

**Fig. 1:**
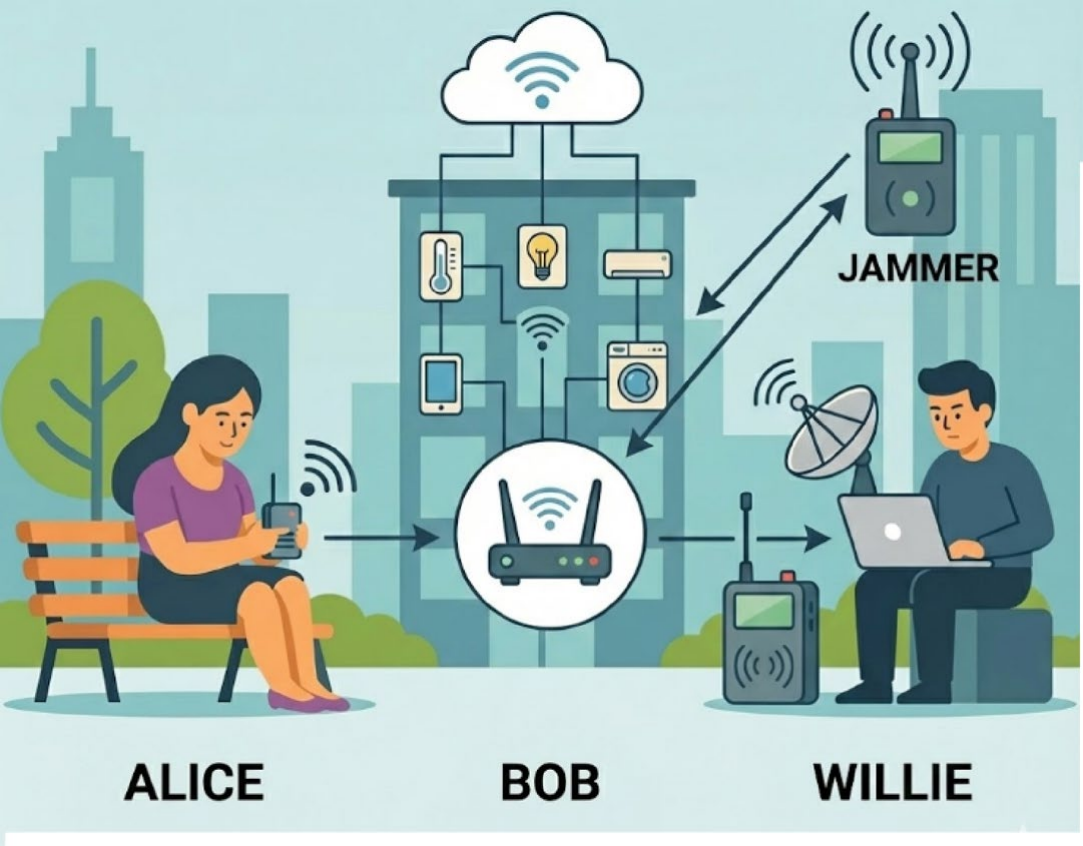
Covert communication system model includes Alice, Bob, Willie, and a friendly jammer.

We consider a system consisting of four entities: Alice (transmitter), Bob (receiver), an eavesdropper or Willie, and a friendly jammer. Alice attempts to transmit information to Bob, while Willie tries to detect whether Alice is active or silent. The friendly jammer continuously transmits artificial noise to confuse Willie, while being designed not to interfere with Bob’s reception, thereby maintaining a consistent background energy level at Willie’s receiver (Fig. 1).

In practical wireless environments, the transmission channel between communicating nodes is subject to multiple impairments that significantly affect signal propagation and detection performance. These impairments include large scale path loss caused by the physical separation of nodes, small scale fading due to multipath propagation, and additive noise originating from background thermal and electronic sources. In addition, intentional or unintentional interference such as that generated by a friendly jammer further alters the received signal statistics at the Willie. Therefore, an accurate channel model must incorporate these effects to realistically represent the covert communication scenario. In this work, we adopt a composite channel model that accounts for both large scale attenuation and small-scale Rayleigh fading, combined with additive white Gaussian noise and external jamming interference, as detailed below. The received signal at Willie during a single transmission slot can be modeled as:

1 $${y}_{w}\left(t\right)={x}_{a}\left(t\right)\frac{{h}_{aw}}{\sqrt{{L(d)}_{aw}}}.\beta +{x}_{j}\left(t\right)\frac{{h}_{jw}}{\sqrt{{L(d)}_{jw}}}+{n}_{w}(t)$$

where ${x}_{a}\left(t\right)$ and ${x}_{j}\left(t\right)$ denote Alice’s and the jammer’s baseband signals respectively. The coefficients ${h}_{aw}$ and ${h}_{jw}$ represent the small-scale fading of the Alice to Willie and jammer to Willie channels, modeled as independent circularly symmetric complex Gaussian random variables $\mathcal{C}\mathcal{N}(\mathrm{0,1})$. The large-scale path loss component between any two nodes separated by distance d is modeled as $L(d)={d}^{\alpha }$ where $\alpha$ is the path loss exponent that characterizes propagation conditions (typically between 2 and 4 in wireless environments). The additive noise ${n}_{w}(t)$ is modeled as complex Gaussian noise ${n}_{w}(t)\sim \mathcal{C}\mathcal{N}(0,{\sigma }^{2})$. The binary parameter $\beta \in \left\{\mathrm{0,1}\right\}$ captures Alice’s transmission activity; that is, $\beta =0$ corresponds to Alice’s transmitter being inactive, while $\beta =1$ corresponds to her transmitter being active.

The jammer is assumed to be friendly, i.e., it affects only Willie’s channel and is designed not to interfere with Bob’s reception. Therefore, the received signal at Bob can be expressed as:

2 $${y}_{b}\left(t\right)={x}_{a}\left(t\right)\frac{{h}_{ab}}{\sqrt{{L(d)}_{ab}}}+{n}_{b}(t)$$

where ${h}_{ab}\sim \mathcal{C}\mathcal{N}(\mathrm{0,1})$ denotes the small-scale fading of Alice to Bob channel, ${L(d)}_{ab}$ represents the corresponding large scale path loss, and ${n}_{b}\left(t\right)\sim \mathcal{C}\mathcal{N}(0,{\sigma }^{2})$ is the additive Gaussian noise at Bob. Importantly, the friendly jammer is assumed to have no impact on Bob’s reception, which guarantees that Alice’s transmission can always be decoded reliably as long as the transmit power satisfies Bob’s minimum Signal to Noise Ratio (SNR) requirement.

It is evident that Willie’s received signal is affected not only by Alice’s potential transmission but also by the artificial interference generated by the jammer. This cooperative jamming strategy significantly increases Willie’s uncertainty in distinguishing between the two hypotheses:

$$\left\{ {\begin{array}{*{20}l} {H_{0} :\beta = 0,\;Alice\;is\;silent\;\left( {only\;jammer + noise} \right)} \hfill \\ {H_{1} :\beta = 1,\;Alice\;is\;active\;\left( {Alice + jammer + noise} \right)} \hfill \\ \end{array} } \right.$$

Willie thus performs a binary hypothesis test between ${H}_{0}$ and ${H}_{1}$ based on his received ${y}_{w}\left(t\right)$.

**Fig. 2:**
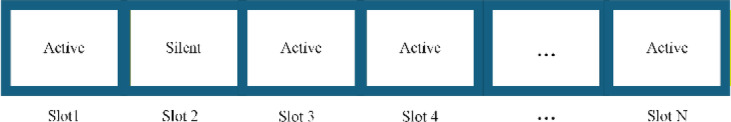
Pseudo-random time-slot transmission pattern used in covert communication.

The covert transmission process is organized into a sequence of discrete time slots, each representing an independent observation interval during which Willie performs his detection operation. Within each slot, all wireless channels including Alice to Willie, jammer to Willie, and Alice to Bob links are assumed to remain constant (quasi static fading), while they change independently from slot to slot to capture realistic temporal variations. To further enhance covertness, Alice adopts a pseudo random transmission pattern, where each slot is independently selected as either active (transmission) or silent according to a uniform random process. This randomness prevents Willie from inferring any deterministic transmission pattern over time, thereby increasing the difficulty of reliable detection (Fig. 2).

This slot-based structure enables both theoretical analysis and simulation-based validation by treating each slot as an independent detection trial. It also facilitates the generation of large, statistically diverse signal realizations, which later serve as labeled data samples for machine learning based covert detection analysis.

## Detection errors and optimal threshold

Willie’s goal is to distinguish whether the received signal corresponds to Alice’s silence or her active transmission state, denoted by the two hypotheses ${H}_{0}$ and ${H}_{1}$, respectively.

Willie computes a scalar test statistic from the received complex baseband samples in each slot in our setup we use the sample average energy

3 $$E=\frac{1}{N}{\sum}_{n=1}^{N}{\left|{y}_{w}\left[n\right]\right|}^{2}$$

where N is the number of complex samples per slot and ${y}_{w}\left[n\right]$ denotes the n the received complex sample at Willie. In binary hypothesis test, Willie faces two types of decision errors: false alarm and missed detection.

A false alarm occurs when Alice is actually silent ${H}_{0}$, yet Willie declares that a transmission is present $H={H}_{1}$.In practice, this means that the detector frequently raises spurious alarms although no covert communication takes place. Conversely, a missed detection happens when Alice is active ${H}_{1}$, but Willie decides in favor of silence $H={H}_{0}$; in this case, the covert signal successfully evades detection.

Willie employs an energy detection to decide whether Alice is transmitting or not. The received signal energy in each observation window is compared against a decision threshold $\gamma$. If the measured energy exceeds this threshold, Willie declares Alice as active; otherwise, he concludes that Alice remains silent. The performance of such a detector is characterized by two probabilities: the false alarm probability and the missed detection probability. The former represents the likelihood of raising an alarm in the absence of any transmission, while the latter quantifies the chance of failing to detect Alice when she is active. Formally, these probabilities can be expressed as:

4 $${P}_{FA}=\mathit{Pr}(E>\gamma |{H}_{0})$$

5 $${P}_{MD}=\mathit{Pr}(E\le \gamma |{H}_{1})$$

Under Rayleigh fading and additive white Gaussian noise, the received energy follows an exponential distribution under both hypotheses. Hence, the closed form expressions of ${P}_{FA}$ and ${P}_{MD}$ are given by^30^:

6 $$P_{FA} = \left\{ {\begin{array}{*{20}l} {exp^{{ - \frac{{\left( {\gamma - \sigma_{w}^{2} } \right)}}{{\mu_{jw} }}}} ,} \hfill & {\gamma - \sigma_{w}^{2} > 0} \hfill \\ {1,} \hfill & {\gamma - \sigma_{w}^{2} \le 0} \hfill \\ \end{array} } \right.$$

7 $$P_{MD} = \begin{array}{*{20}l} {1 + \frac{{ - \mu_{aw} exp^{{ - \frac{{\left( {\gamma - \sigma_{w}^{2} } \right)}}{{\mu_{aw} }}}} + \mu_{jw} exp^{{ - \frac{{\left( {\gamma - \sigma_{w}^{2} } \right)}}{{\mu_{jw} }}}} }}{{\mu_{aw} - \mu_{jw} }},} \hfill & {\gamma - \sigma_{w}^{2} > 0} \hfill \\ 0 \hfill & {\gamma - \sigma_{w}^{2} \le 0} \hfill \\ \end{array}$$

where ${P}_{a}$and ${P}_{j}$denote the transmit powers of Alice and the jammer, respectively; ${\mu}_{aw}={P}_{a}/{L}_{aw}$ and ${\mu}_{jw}={P}_{j}/{L}_{jw}$ represent the corresponding average received powers at Willie; and ${\sigma }^{2}$ is the background noise power.

These two error probabilities are closely related to the choice of the detection threshold. Increasing the threshold suppresses false alarms by making the detector more conservative, but it simultaneously raises the likelihood of missed detections. Conversely, lowering the threshold reduces missed detections at the cost of more frequent false alarms. Therefore, the threshold plays a central role in balancing these two opposing errors. Since no single threshold can minimize both ${P}_{FA}$ and ${P}_{MD}$ simultaneously, Willie seeks an optimal trade off that minimizes their total sum, i.e., ${P}_{FA}+{P}_{MD}$.

According to classical detection theory, the optimal threshold can be derived in closed form as a function of Alice’s and jammer’s received average powers, denoted by ${\mu}_{aw}$ and ${\mu}_{jw}$ respectively, as well as the background noise level ${\sigma}_{w}^{2}$:

8 $${\gamma }^{*}=\frac{{\mu}_{aw}{\mu}_{jw}}{{\mu}_{jw}-{\mu}_{aw}}\mathit{ln}(\frac{{\mu}_{aw}}{{\mu}_{jw}})+{\sigma}_{w}^{2}$$

The resulting threshold expression ensures that the total error probability, i.e., ${P}_{FA}+{P}_{MD}$, is minimized. Detailed derivations of this expression can be found in^31^, while here we directly adopt the closed form solution for subsequent analysis.

## The proposed machine learning based covert communication detection

In contrast to conventional energy detection approaches, which rely on a single scalar test statistic and a fixed or analytically optimized threshold, the proposed framework reformulates the covert detection problem as a supervised signal classification task in a high-dimensional feature space. From Willie’s perspective, the objective is no longer to compare the received energy against a predefined threshold, but to infer the latent transmission state of Alice based on the statistical structure of the received waveform under uncertainty.

The proposed detection framework is decomposed into two main stages: (1) baseband feature extraction from the received signal, and (2) supervised learning–based decision making and performance evaluation.

**Fig. 3:**
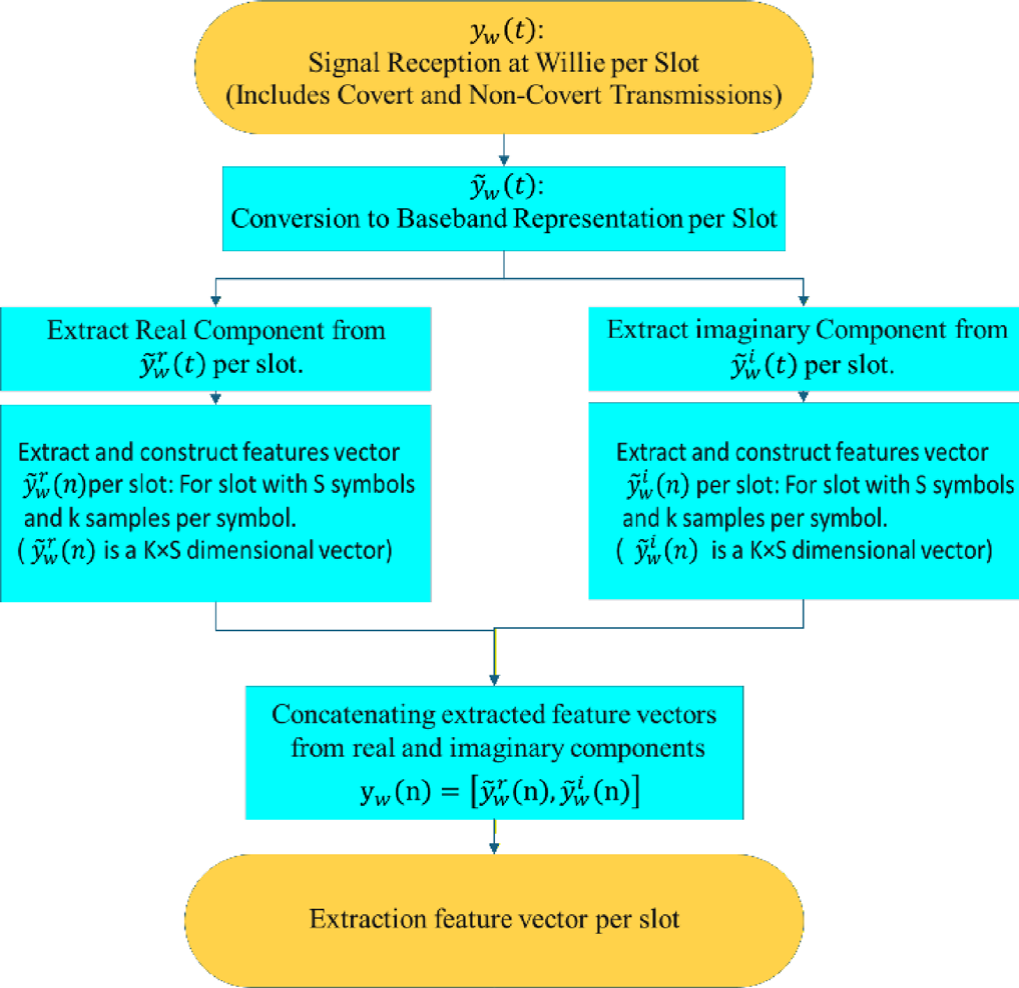
The proposed feature extraction process at Willie per time slot.

Steps of the proposed baseband feature extraction method are given in Fig. 3.

In each observation slot, Willie observes the received signal ${y}_{w}\left(t\right)$, as defined in "System and channel model" section, which consists of a superposition of Alice’s covert transmission (when active), jamming interference, multipath fading effects, and additive noise. Without loss of generality, the received waveform is directly modeled in the complex baseband domain and denoted by ${\widetilde{y}}_{w}\left(t\right)$. This baseband representation preserves both amplitude and phase information of the received signal, which are essential for characterizing covert transmission behavior under fading and jamming uncertainty. To enable effective processing of the received baseband signal, it is necessary to further decompose the complex waveform into its fundamental in-phase and quadrature components, which provide complementary information about the underlying transmission characteristics.

Following the baseband signal representation, the in-phase and quadrature components of the received signal, denoted by ${\widetilde{y}}_{w}^{r}\left(t\right)$ and ${\widetilde{y}}_{w}^{i}(t)$, are extracted separately for each observation slot. These components jointly capture instantaneous energy fluctuations as well as phase distortions introduced by multipath fading and jamming. Processing the signal in this form enables the detector to implicitly exploit energy-related information traditionally used by energy-based detectors, while simultaneously preserving phase information that is discarded in conventional energy detection. While the continuous-time in-phase and quadrature components capture rich signal information, they must be transformed into a structured numerical representation to serve as suitable inputs for the subsequent learning-based detection stage. While the continuous-time in-phase and quadrature components capture rich signal information, they must be transformed into a structured numerical representation to serve as suitable inputs for the subsequent learning-based detection stage.

For a slot containing S transmitted symbols and k samples per symbol, each component is reshaped into a feature vector of length k × S. After sampling and slot-wise segmentation, the extracted components corresponding to the n-th observation slot are represented as discrete vectors.

9 $$\tilde{y}_{w}^{r} \left( n \right) = [\tilde{y}_{w}^{r} \;(1),\;\tilde{y}_{w}^{r} \;(2), \ldots ,\tilde{y}_{w}^{r} \;({\mathrm{k}} \times {\mathrm{S}})]$$

10 $$\tilde{y}_{w}^{i} \left( n \right) = [\tilde{y}_{w}^{i} \;(1),\;\tilde{y}_{w}^{i} \;(2), \ldots \tilde{y}_{w}^{i} \;({\mathrm{k}} \times {\mathrm{S}})]$$

This slot-based representation preserves the temporal structure of the received signal and enables the learning model to observe fine-grained fluctuations across symbols and samples. Such fluctuations are particularly informative in covert communication scenarios, where transmission power is deliberately kept low and statistical differences are subtle.

To jointly exploit the amplitude and phase information, the real and imaginary feature vectors are concatenated to form the final input representation as:

11 $${\mathrm{y}}_{w}\left(\mathrm{n}\right)=\left[{\widetilde{y}}_{w}^{r}\left(\mathrm{n}\right),{\widetilde{y}}_{w}^{i}\left(\mathrm{n}\right)\right]$$

resulting in a unified representation that jointly captures amplitude- and phase-related characteristics of the received signal. This feature vector constitutes the sole input to the subsequent detection framework, providing a direct and physically meaningful interface between the wireless channel and the data-driven learning stage.

By using the real and imaginary components of the complex baseband signal as input features, the learning model implicitly captures both amplitude and phase variations introduced by fading and jamming. These features preserve characteristics that are typically ignored or averaged out in energy-only detectors. As a result, the classifier can exploit subtle differences in signal structure that are not accessible through scalar energy metrics alone.

**Fig. 4:**
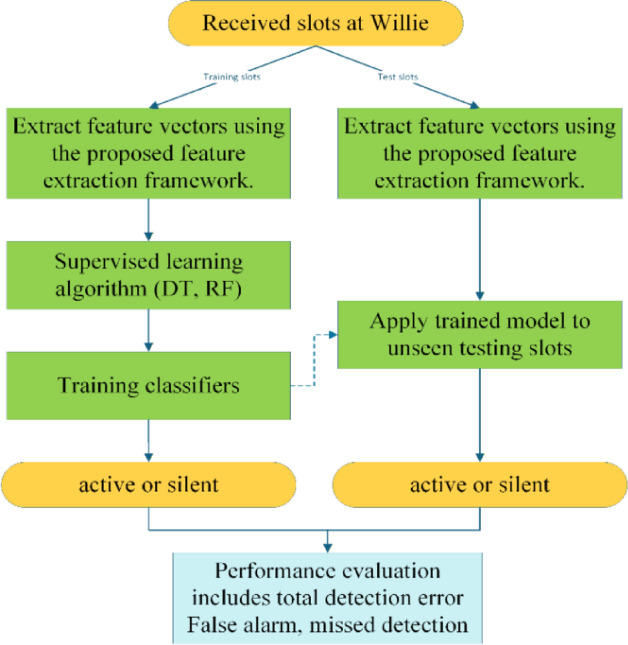
The framework of the proposed machine learning–based covert communication detection.

In the following, the framework of the proposed machine learning–based covert communication detection is given in Fig. 4.

A systematic learning and decision-making framework is required to map these features to the underlying transmission state of Alice. To this end, the proposed approach integrates the extracted baseband features into a supervised machine learning pipeline that enables training, test, and objective performance evaluation under fading and jamming uncertainty.

In the training phase, feature vectors corresponding to the training slots are processed using supervised machine learning algorithms, specifically Decision Tree and Random Forest classifiers. Each training sample is associated with a known transmission label indicating whether Alice is active (covert transmission) or silent during the corresponding time slot. By exploiting the extracted baseband features, the learning algorithms iteratively construct decision rules that capture the combined effects of fading, jamming interference, and noise. Unlike conventional energy detection that rely on fixed thresholds and prior knowledge of signal power levels, the trained models learn adaptive and nonlinear decision boundaries directly from the observed data.

The trained classifier is applied to the test phase, where feature vectors extracted from previously unseen test slots are used as inputs. In this phase, no label information is provided to the model, and the classifier independently predicts the transmission state of Alice for each time slot. This test procedure emulates a realistic monitoring scenario in which Willie must make detection decisions based solely on real-time signal observations without access to system parameters or ground-truth transmission states. The predicted decisions from the test slots are then used to evaluate the performance of the proposed detection framework.

In the proposed framework, the trained ML classifier can be viewed as constructing a multidimensional, adaptive decision region that replaces the conventional fixed threshold. This decision region dynamically accounts for the joint effects of channel fading, interference, and noise, enabling reliable detection even when the instantaneous SNR fluctuates significantly. Importantly, this adaptive behavior emerges solely from data-driven learning, without requiring prior knowledge of Alice’s transmit power, jammer power, or channel statistics. It should be clarified that since the model is trained on data generated under specific static conditions, it learns to distinguish states based on the structure of the received real and imaginary features. Consequently, during the inference phase, the detector operates in a power-agnostic manner it does not require the exact numerical values of or as inputs. This property is particularly valuable in large-scale IoT environments, where devices operate with heterogeneous power levels, sporadic transmission patterns, and unpredictable interference sources. In such networks, maintaining accurate parameter estimates for analytical detectors is often impractical. By contrast, the proposed ML-based detector operates in a power-agnostic manner, making it well suited for real-world monitoring scenarios where Willie observes a mixture of legitimate IoT traffic and potentially covert transmissions. Furthermore, the inclusion of a friendly jammer in the system model introduces an additional layer of uncertainty that is difficult to characterize analytically. While classical detectors typically suffer performance degradation under strong jamming, the proposed learning-based framework leverages the jammer-induced randomness as part of the training data. This enables the classifier to learn robust decision rules that remain effective across a wide range of jamming power levels, as confirmed by the simulation results.

Overall, the proposed framework transforms covert communication detection from a parameter-dependent hypothesis test problem into a robust data-driven inference task. By tightly integrating physical-layer signal modeling with supervised learning, the framework provides a flexible and scalable solution for monitoring covert activity in interference-limited and dynamically varying wireless environments, such as those encountered in modern IoT networks.

## Experiments and results

To evaluate the performance of the machine learning-based detection, a series of experiments were conducted under various channel and interference conditions. The received signals were generated using the same system and channel parameters described earlier, with the Alice to Willie separation set to distances ranging from 1 to 5 km. These distance values represent realistic transmission ranges used to control the path loss effect. For each scenario, signal samples corresponding to both active ($\beta =1$) and silent ($\beta =0$) transmission states were generated to form a balanced dataset suitable for supervised learning. The complex baseband signals were processed in MATLAB to extract their real and imaginary components, which were then directly used as input data for the ML models without explicit feature extraction.

### Simulation setup

The analytical and simulation-based detectors were implemented in MATLAB, while the ML based classifiers were developed in Python using the Scikit learn library. The simulations were carried out under a Rayleigh fading channel with additive white Gaussian noise. The main system and simulation parameters are summarized as follows: the number of transmission slots was set to ${N}_{slot}={20,000}$, and each slot contained S = 100 Binary Phase Shift Keying (BPSK) modulated bits. The transmit powers of Alice and the jammer were ${P}_{A}=2 \,\mathrm{and}\, {P}_{J}=10$, respectively, while the background noise power was fixed at ${\sigma }^{2}={10}^{-6}$. A Raised Cosine transmit filter was used with a roll off factor of 0.35, a filter span of 10 symbols, and k = 8 samples per symbol. The path loss model was defined as $L(d)={d}^{2}$, and transmitter–receiver distances were varied from 1 to 5 km. The signal to noise ratio threshold for reliable reception at Bob was set to 6 dB, and the path loss range satisfying this condition was selected for simulation. Each result was averaged over 100 independent Monte Carlo realizations to ensure statistical reliability. For every distance scenario, both active ($\beta =1$) and silent ($\beta =0$) transmission patterns were randomly generated to represent covert communication slots. The received baseband signal at Willie was modeled as the superposition of Alice’s and jammer’s signals plus background noise. For each distance scenario, both theoretical analysis and Monte Carlo simulations were performed to evaluate the false alarm probability and missed detection probability of the Willie’s detector. Each result was averaged over 100 independent realizations to ensure statistical reliability. The obtained probabilities were finally plotted as functions of the distance (in km) between Alice and Willie to illustrate the impact of path loss and fading on covert communication detectability.

### Simulation results and discussion

**Fig. 5:**
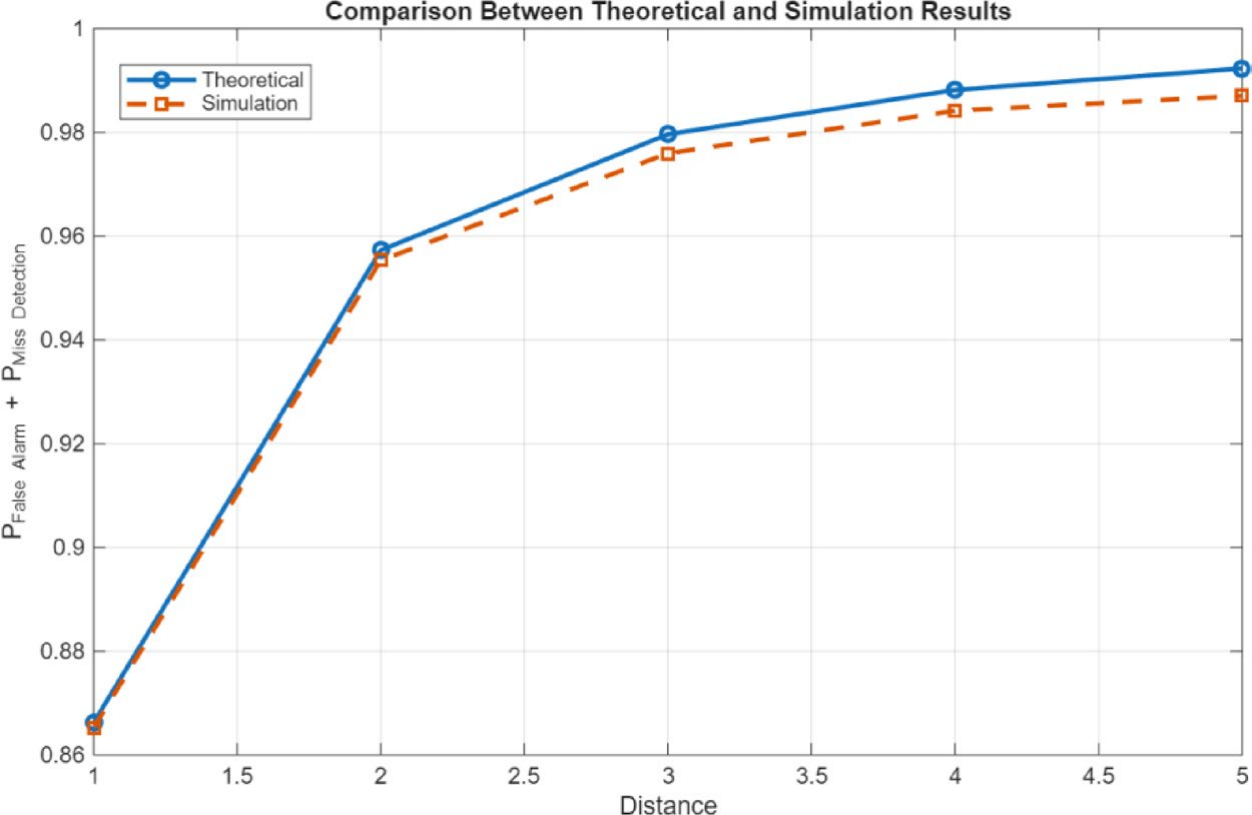
Comparison between analytical and Monte Carlo simulation results for the simulation-based model under jamming interference.

The performance of both the analytical simulation models and the machine learning-based detection framework was evaluated under varying distances between Alice and Willie, ranging from $d=1$ to $5$ km. Figure 5 present the false alarm probability, missed detection probability, and the total detection error (${P}_{FA}+{P}_{MD}$) obtained from theoretical analysis, Monte Carlo simulations, and the trained machine learning classifiers.

At shorter distances, where the received power at Willie is relatively strong, the analytical and simulation results exhibit close agreement, confirming the correctness of the theoretical derivations. However, as the distance increases, fading and jamming introduce significant randomness in the received energy, causing small deviations between the analytical and simulated curves. This deviation mainly originates from the idealized assumptions in the analytical model, which neglect instantaneous variations in channel coefficients.

Before evaluating the machine learning based detectors, a confusion matrix analysis was performed to interpret the detection outcomes in the context of covert communication. Each entry of the confusion matrix corresponds to a possible decision made by the classifier with respect to Alice’s actual transmission state. In this representation, the vertical axis corresponds to the actual transmission state of Alice (Active or Silent), while the horizontal axis represents the predicted state determined by the detector.

**Fig. 6:**
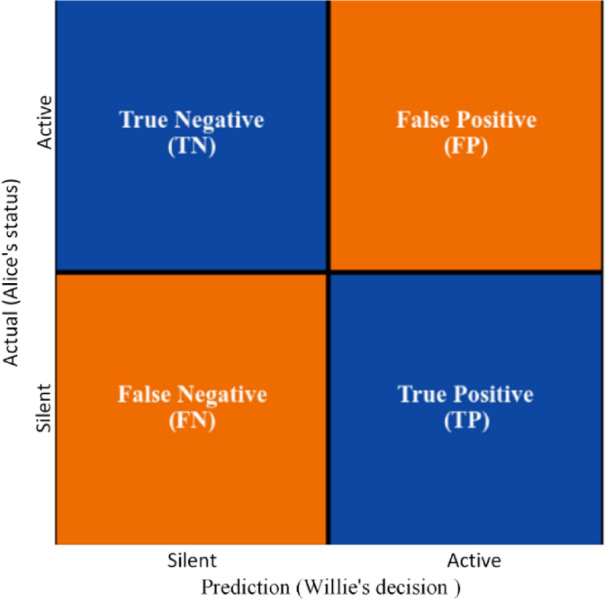
Confusion matrix for covert communication detection, showing the relationship between the actual transmission state of Alice and the predicted state determined by the detector.

This orientation, as shown in Fig. 6, allows a direct comparison between the true and predicted classes, facilitating the identification of correct detections and classification errors. Specifically, a true positive (TP) indicates that Alice was transmitting and the detector correctly identified the active state; a true negative (TN) means Alice was silent and the detector correctly recognized the silence; a false positive (FP) represents a false alarm, where the detector incorrectly declares transmission during a silent slot; and a false negative (FN) corresponds to a missed detection, where the detector fails to detect an ongoing transmission.

Based on these outcomes, the false alarm probability and missed detection probability are computed as:

11 $${P}_{FA}=\frac{FP}{TN+FP}$$

12 $${P}_{MD}=\frac{FN}{TP+FN}$$

These two probabilities together characterize the detection performance of the Willie, and their sum (${P}_{FA}+{P}_{MD}$) represents the total detection error used as the main evaluation metric in this study.

**Fig. 7:**
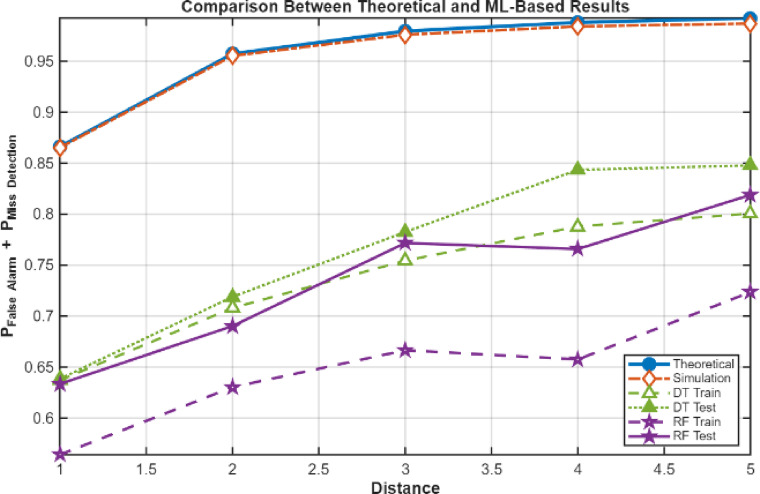
Total detection error of Willie versus all distance for analytical, simulation-based, and the proposed machine learning–based detectors for $P_{A} = 2{ }$ and $P_{J} = 10$.

The machine learning-based detectors specifically the Decision Tree and Random Forest were then evaluated using the simulated signal datasets. Figure 7 illustrates the comparison between theoretical, simulation based, and ML based results for the total detection error as a function of distance. The results clearly indicate that both ML models outperform the analytical and simulation-based detectors. The Decision Tree model achieves moderate improvement, reducing the total detection error to approximately 0.64–0.84 across the distances tested. In contrast, the Random Forest model consistently achieves the lowest total error, with values ranging from approximately 0.63 at $d=1$ km to 0.81 at $d=5$ km.

This superior performance of the Random Forest model can be attributed to its ensemble learning structure, which combines multiple decision trees to reduce variance and improve robustness under random fading and interference. As the distance increases, all detectors experience an increase in ${P}_{FA}+{P}_{MD}$, reflecting the reduced signal power and higher uncertainty at the Willie. However, the Random Forest classifier maintains greater stability and accuracy, achieving superior performance even at longer distances. These findings highlight the potential of machine learning techniques to enhance covert communication analysis, particularly in challenging conditions such as fading and jamming.

At the shortest distance ($d=1 \,\mathrm{km}$), a direct numerical comparison highlights the advantage of the machine learning-based over the simulation-based detector. The total detection error of the conventional simulation model is approximately 0.8653, whereas the Decision Tree and Random Forest models achieve total errors of 0.6384 and 0.6334, respectively. These results correspond to performance improvements of about 26.2% for the Decision Tree and 26.8% for the Random Forest compared to the baseline simulation model. This significant reduction in detection error at close range further demonstrates the capability of the ML based framework to learn discriminative patterns from the signal data and to outperform analytical threshold-based detectors even in high SNR conditions. Tables 2 and 3 detail the hyperparameter configurations used for the Decision Tree classifier and Random Forest, respectively.

**Table 2:** Hyperparameters for the Decision Tree classifier across different distance scenarios.

Parameter	Criterion	Max depth	Min samples split	Random state	The number of training samples	The number of test samples
Distance 1–5(km)	Entropy	5	10	42	16,000 slots (80%)	4000 slots (20%)

**Table 3:** Hyperparameters for the Random Forest classifier across different distance scenarios.

Parameter	Max depth	Min samples split	Min samples leaf	N estimators	Max features	Bootstrap	Random state	The number of training samples	The number of test samples
Distance 1–5 (km)	2	10	5	200	sqrt	True	42	16,000 slots (80%)	4000 slots (20%)

**Fig. 8:**
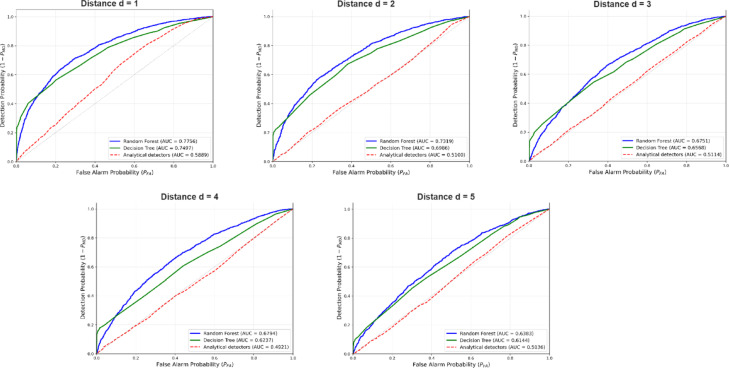
ROC curves comparing the detection performance of DT, RF, and the analytical detector across distances from 1 to 5 km.

To provide a comprehensive evaluation of the detection capabilities and to validate the robustness of the proposed framework across varying network topologies, Receiver Operating Characteristic (ROC) curves were generated for distances ranging from $d=1 \,\mathrm{km}$ to $d=5 \,\mathrm{km}$. Figure 8 illustrates the performance comparison between the Decision Tree, Random Forest, and the conventional analytical threshold-based detector.

A quantitative analysis of the Area Under the Curve (AUC) values, as summarized in Fig. 8, reveals distinct performance tiers among the competing methods. The Random Forest classifier consistently achieves the highest AUC scores across all tested distances, with values of 0.7756, 0.7319, 0.6751, 0.6794, and 0.6383 for $d=1 \,\mathrm{km}$ to $d=5 \,\mathrm{km}$, respectively. This represents a significant improvement over the analytical baseline, which yields AUCs of only 0.5889, 0.5100, 0.5114, 0.4921, and 0.5036 for the same distances. Notably, at $d=4 \,\mathrm{km}$ and $d=5 \,\mathrm{km}$, the analytical detector’s AUC drops near 0.5, indicating a performance no better than random guessing due to the severe path loss and fading effects. In contrast, the RF model maintains an AUC well above 0.6 even at the farthest distance, confirming its ability to extract reliable features from weak signals.

The Decision Tree classifier also outperforms the analytical method, achieving AUCs of 0.7497, 0.6986, 0.6568, 0.6237, and 0.6144. However, the RF model consistently surpasses the DT, particularly at closer ranges (e.g., 0.7756 vs. 0.7497 at $d=1 \,\mathrm{km}$), highlighting the benefits of the ensemble learning approach in reducing variance and improving generalization. These results conclusively demonstrate that the proposed ML-based framework offers a superior and more reliable detection solution for covert communication in large-scale communication networks compared to traditional energy-based techniques.

**Fig. 9:**
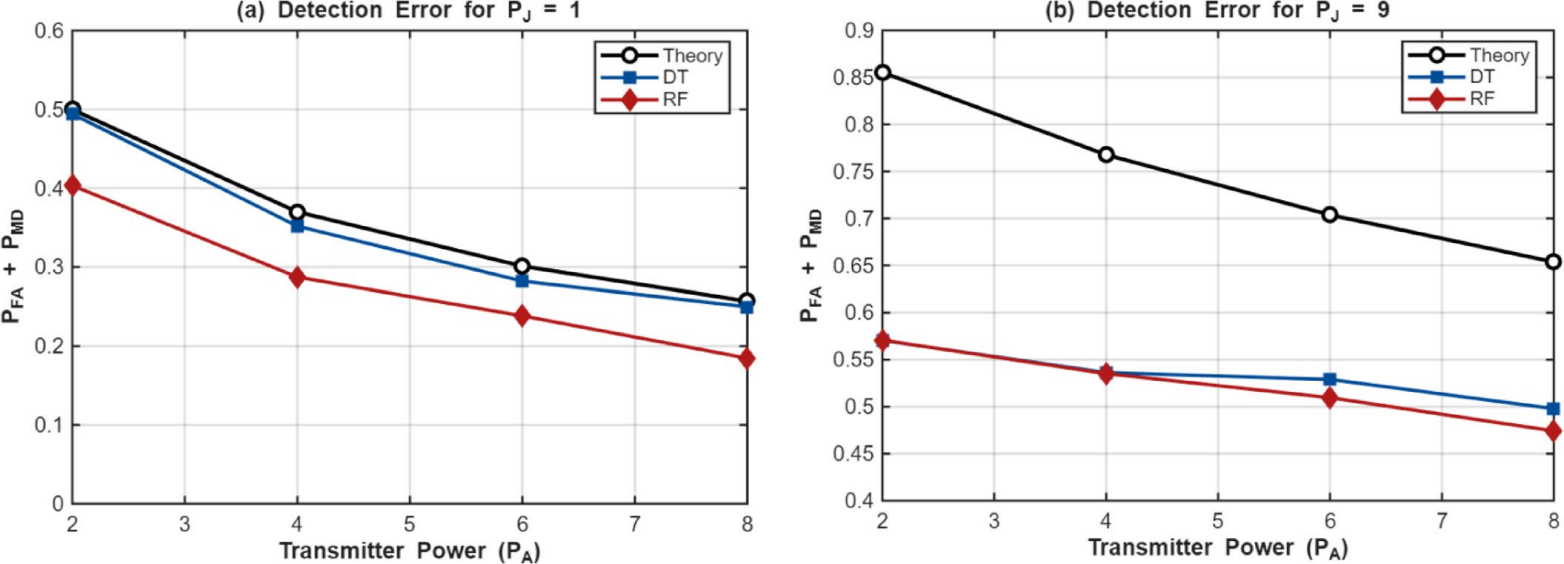
Total detection error of Willie versus different transmitter power values for (**a**) ${P}_{J}=1$ and (**b**) ${P}_{J}=9$ at distance *d* = 1 km

In addition, Fig. 9 compares the theoretical detector with the two ML-based classifiers (DT and RF) under low and high jammer power conditions $({P}_{J}=1 \,\mathrm{and}\, 9)$. All results correspond to a fixed Alice to Willie distance ($d=1 \,\mathrm{km}$), ensuring that the observed variations are solely due to changes in the transmitter and jammer power levels. As expected, the theoretical detector yields the highest total detection error across all ${P}_{A}$ values, with its performance degrading sharply under strong jamming.

In contrast, both ML based classifiers exhibit substantially lower error levels, demonstrating their ability to learn discriminative patterns that are not captured by the analytical threshold model. Under low jamming power, the Decision Tree and Random Forest curves follow a similar trend, while RF consistently maintains a slight advantage due to its more stable ensemble-based decision structure. When the jammer power increases, this advantage becomes more pronounced, highlighting the superior robustness of RF in interference-dominated scenarios. Two general trends emerge from the results. First, increasing the transmitter power ${P}_{A}$ leads to a steady reduction in the total detection error for all detectors, since the received signal becomes easier to distinguish from the interference background. Second, the Random Forest classifier achieves the lowest error across both jamming regimes, confirming its resilience to fading, noise, and strong jamming. Overall, the results demonstrate the clear benefit of data-driven detection methods over analytical energy-based models in realistic wireless environments. The hyperparameter settings for the DT and RF models across the various power configurations are listed in Tables 4 and 5, respectively.

**Table 4:** Configuration of the Decision Tree classifier for different power scenarios at distance $d=1 \,\mathrm{km}$.

Parameter	${{\boldsymbol{P}}}_{{\boldsymbol{J}}}=1$				${{\boldsymbol{P}}}_{{\boldsymbol{J}}}=9$			
	${P}_{a}=2$	${P}_{a}=4$	${P}_{a}=6$	${P}_{a}=8$	${P}_{a}=2$	${P}_{a}=4$	${P}_{a}=6$	${P}_{a}=8$
Criterion	Entropy	Entropy	Entropy	Entropy	Entropy	Entropy	Entropy	Entropy
Max Depth	11	14	17	19	7	7	7	12
Min Samples Split	10	10	10	10	10	10	10	10
Random State	42	42	42	42	42	42	42	42
The number of Training samples	16,000 slots (80%)	16,000 slots (80%)	16,000 slots (80%)	16,000 slots (80%)	16,000 slots (80%)	16,000 slots (80%)	16,000 slots (80%)	16,000 slots (80%)
The number of Test samples	4000 slots (20%)	4000 slots (20%)	4000 slots (20%)	4000 slots (20%)	4000 slots (20%)	4000 slots (20%)	4000 slots (20%)	4000 slots (20%)

**Table 5:** Configuration of the Random Forest classifier for different power scenarios at distance $d=1\, \mathrm{km}$.

Parameter	${P}_{J}=1$				${P}_{J}=9$			
	${P}_{a}=2$	${P}_{a}=4$	${P}_{a}=6$	${P}_{a}=8$	${P}_{a}=2$	${P}_{a}=4$	${P}_{a}=6$	${P}_{a}=8$
Max Depth	8	10	10	13	3	4	4	7
Min Samples Split	10	10	10	10	10	10	10	10
Min Samples Leaf	5	5	5	5	5	5	5	5
N Estimators	200	200	200	200	200	200	200	200
Max Features	sqrt	Sqrt	Sqrt	Sqrt	Sqrt	Sqrt	Sqrt	sqrt
Bootstrap	True	True	True	True	True	True	True	True
Random State	42	42	42	42	42	42	42	42
The number of Training samples	16,000 slots (80%)	16,000 slots (80%)	16,000 slots (80%)	16,000 slots (80%)	16,000 slots (80%)	16,000 slots (80%)	16,000 slots (80%)	16,000 slots (80%)
The number of Test samples	4000 slots (20%)	4000 slots (20%)	4000 slots (20%)	4000 slots (20%)	4000 slots (20%)	4000 slots (20%)	4000 slots (20%)	4000 slots (20%)

**Fig. 10:**
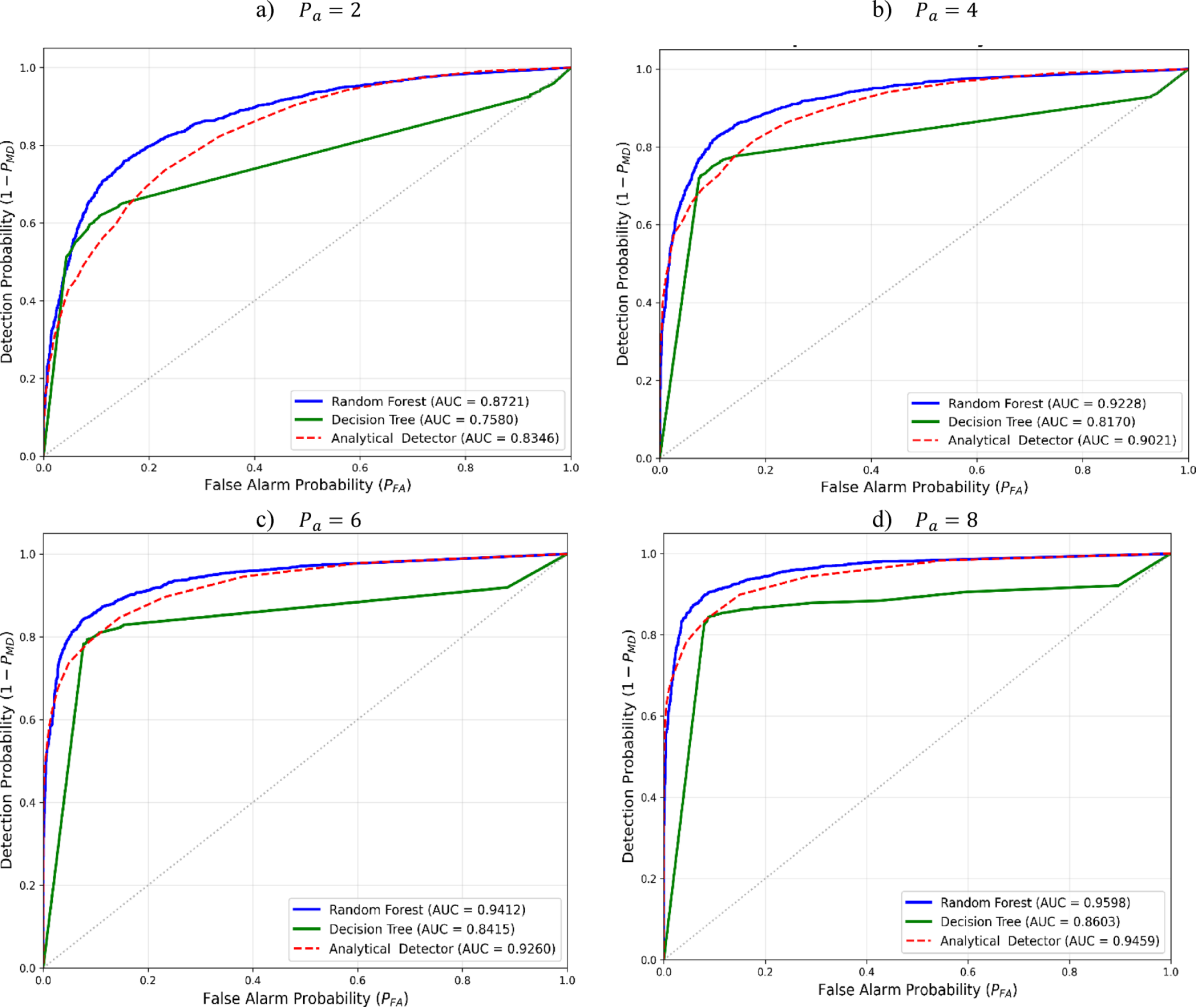
ROC curves under low jamming conditions (${P}_{J}=1$). Subplots (**a**)–(**d**) correspond to transmit power levels ${P}_{a}=2, 4, 6, \mathrm{a}\mathrm{n}\mathrm{d} 8$, respectively. The performance of RF, DT, and analytical detectors is compared in terms of AUC.

**Fig. 11:**
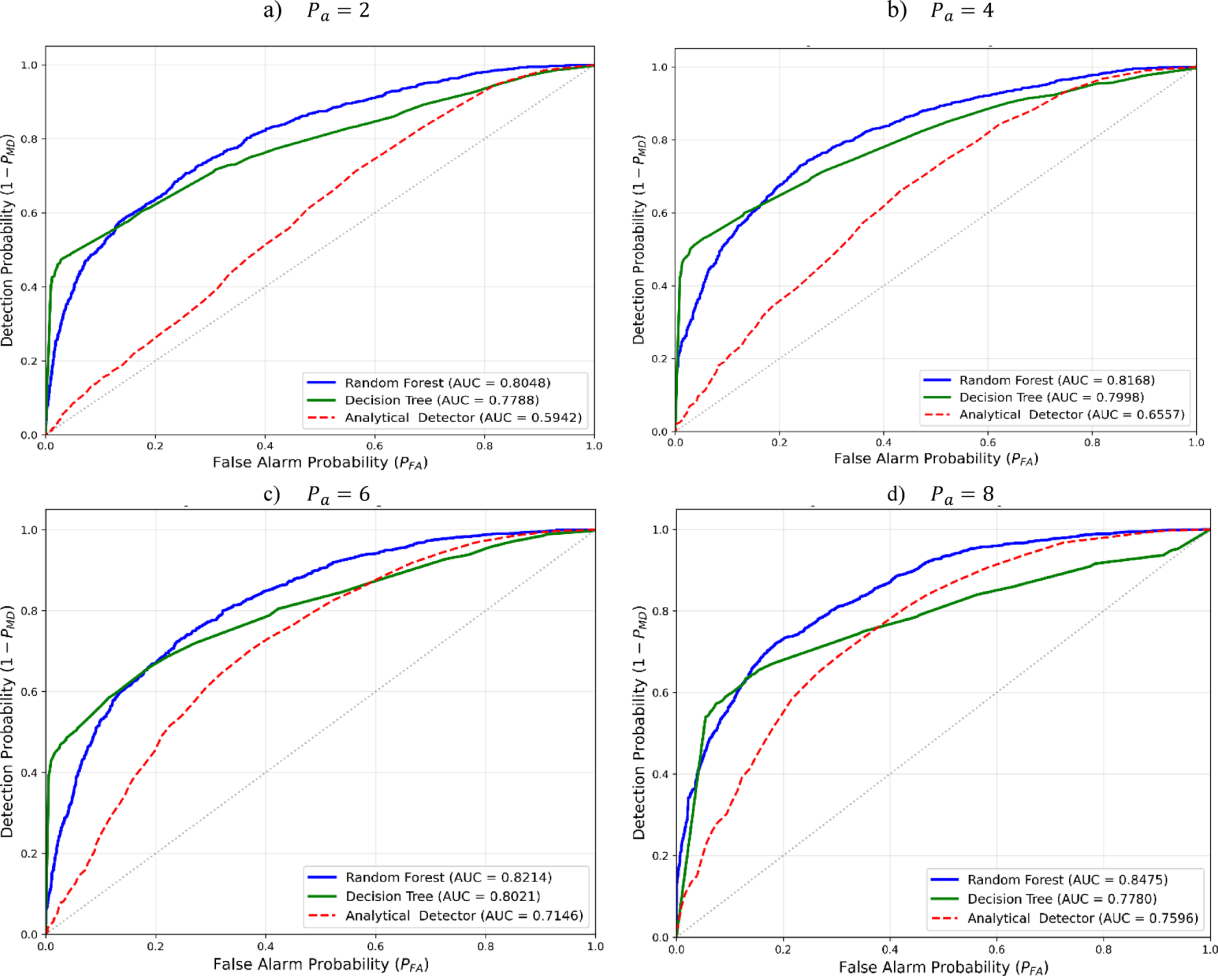
ROC curves under high jamming conditions (${P}_{J}=9$). Subplots (**a**)–(**d**) correspond to transmit power levels ${P}_{a}=2, 4, 6, \mathrm{a}\mathrm{n}\mathrm{d} 8$, respectively. The performance of RF, DT, and analytical detectors is compared in terms of AUC.

The ROC curves presented in Figs. 10 and 11 provide a detailed comparison of detection performance across different transmit power levels under low and high jamming conditions. In both figures, subplots (a)–(d) correspond to transmit power levels ${P}_{a}=2, 4, 6,$ and $8$, respectively. A consistent observation across all subplots is the superior performance of the Random Forest classifier relative to the Decision Tree (DT) and analytical detectors.

Under low jamming conditions (${P}_{J}=1$), all three methods achieve relatively high AUC values, as illustrated in Fig. 10a–d. The analytical detector performs competitively, particularly at lower transmit powers (e.g., Fig. 10a). However, RF consistently achieves the highest AUC across all ${P}_{a}$ values, improving from 0.8721 in Fig. 10a to 0.9598 in Fig. 10d. While the analytical method follows a similar trend, its performance remains slightly below RF, indicating that ensemble learning provides a measurable gain even in less uncertain environments. In contrast, DT exhibits the lowest AUC values across all subplots, reflecting its limited capacity to capture subtle variations compared to RF.

A more pronounced distinction emerges under strong jamming conditions (${P}_{J}=9$), as shown in Fig. 11a–d. In this challenging scenario, the analytical detector suffers a significant performance degradation, with AUC values dropping sharply (e.g., 0.5942 in Fig. 11a). Although DT demonstrates improved robustness compared to the analytical approach, its performance remains moderate and slightly inconsistent across subplots.

In contrast, RF maintains a clear performance advantage across all transmit power levels, achieving AUC values in the range of 0.8048 to 0.8475 in Fig. 11a–d. This indicates that RF is significantly more resilient to the uncertainty introduced by strong jamming. The ensemble structure of RF, which aggregates multiple decision trees, enables it to better capture complex and nonlinear relationships in the received signal, resulting in improved generalization under adverse conditions.

In summary, while analytical detectors achieve acceptable performance under ideal or low-interference conditions, their effectiveness degrades significantly as environmental uncertainty and jamming power increase. In contrast, the proposed machine learning–based approach, particularly the Random Forest model, demonstrates superior robustness and maintains reliable detection performance under more challenging conditions. This indicates that, unlike analytical methods relying on simplified assumptions, learning-based models can better capture the complex and nonlinear effects induced by strong jamming and channel variations. Therefore, the proposed method is better suited for practical covert communication scenarios where harsh and unpredictable conditions are unavoidable.

### Interpretability analysis and feature contribution

To provide transparency into the black-box decision-making process of the proposed Random Forest classifier, we conducted a comprehensive feature importance analysis. Given that the input vector comprises 1600 raw samples representing the complex baseband signal, we aggregated the importance scores into two primary feature groups: the Real component and the Imaginary component By aggregating the importance scores, we can determine whether the model relies on specific signal dimensions or if it requires the complete complex representation to distinguish between transmission states under uncertainty.

To rigorously assess the individual contribution of each signal component, we performed a systematic ablation study. In this experiment, rather than training the models on the complete 1600-dimensional vector (which includes 800 real sampled components concatenated with 800 imaginary sampled components per slot), we constructed two distinct feature subsets to isolate the impact of each domain. First, we sliced the original dataset to retain only the first 800 features corresponding to the Real part, creating a “Real-only” feature set. Second, we isolated the last 800 features corresponding to the Imaginary part to form an “Imag-only” feature set. Finally, we utilized the original concatenated vector as the “Full” feature set. We then trained and evaluated the Decision Tree and Random Forest classifiers independently on these three subsets under identical hyperparameter settings and channel conditions. This isolation technique allows us to directly measure the performance degradation (or improvement) resulting from the removal of specific signal information, thereby quantifying the relative importance of the in-phase and quadrature components.

**Fig. 12:**
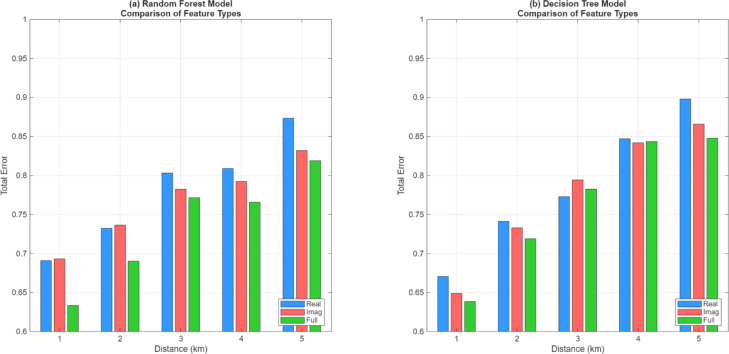
Ablation study results showing total detection error for different feature sets. (**a**) Random Forest classifier. (**b**) Decision Tree classifier. The “Full” feature set (concatenation of Real and Imaginary parts) achieves the lowest error rate in both cases.

The results of this study, summarized in Fig. 12, illustrate the Total Detection Error across the different configurations. The evaluation demonstrates that while the models can operate using individual components, they achieve optimal performance only when the Full feature set is utilized. Specifically, for the Random Forest classifier, the total error decreases from 0.6902 (Real only) and 0.7324 (Imag only) to 0.6334 when both components are combined. A similar trend is observed for the Decision Tree, where the Full feature set reduces the error to 0.6384, outperforming the Real-only (0.7413) and Imag-only (0.7330) scenarios. This significant reduction in error confirms that both the in-phase and quadrature components carry unique and complementary information. Consequently, the proposed framework effectively leverages the complex interplay between amplitude and phase, providing a robust detection capability that surpasses single-dimensional feature extraction methods.

## Conclusion

In this paper, a machine learning–based detection framework was developed to address the problem of covert communication detection in wireless environments affected by fading and jamming interference. By reformulating the detection task as a supervised classification problem, the proposed approach moves beyond conventional energy-based hypothesis testing and enables adaptive decision-making directly from raw signal observations.

The experimental results demonstrated that both Decision Tree and Random Forest classifiers significantly outperform traditional analytical and simulation-based detectors in terms of total detection error and AUC performance. In particular, the Random Forest model consistently achieved the best results across all evaluated scenarios, reducing the total detection error by up to 26.8% compared to the baseline at short distances.

A key finding of this work is that the performance gap between analytical and learning-based methods becomes more pronounced as the environment becomes more challenging. Under strong jamming conditions and increased channel uncertainty, the analytical detector experiences substantial performance degradation due to its reliance on simplified statistical assumptions and precise parameter knowledge. In contrast, the proposed machine learning–based framework maintains stable and reliable performance, demonstrating strong robustness against interference, fading, and power variability. This behavior highlights a fundamental advantage of data-driven approaches: the ability to learn complex and nonlinear signal structures directly from observations, without requiring prior knowledge of transmit power, jammer power, or channel statistics. As a result, the proposed method operates in a power-agnostic manner, making it particularly suitable for large-scale and heterogeneous wireless networks such as IoT systems.

Overall, the results confirm that while analytical detectors may remain effective under ideal or mildly impaired conditions, they are not sufficient for realistic environments characterized by uncertainty and interference. The proposed framework provides a scalable, adaptive, and robust alternative that is better aligned with practical deployment scenarios in modern wireless networks.

Future work will focus on enhancing the generalization capability of the model under dynamic and non-stationary environments, including varying power distributions, mobility, and real-world signal measurements. In addition, extending the framework to deep learning architectures and online learning mechanisms represents a promising direction for further improving detection performance in highly complex scenarios. We also plan to incorporate software-defined radio (SDR) testbeds in future research to address practical implementation challenges and validate the proposed system under real-world operating conditions.

## Data Availability

The datasets generated and analyzed during the current study are available from the corresponding author (R.H.) on reasonable request.
